# SerpinB3 upregulates the Cyclooxygenase-2 / β-Catenin positive loop in colorectal cancer

**DOI:** 10.18632/oncotarget.14997

**Published:** 2017-02-02

**Authors:** Liliana Terrin, Marco Agostini, Mariagrazia Ruvoletto, Andrea Martini, Salvatore Pucciarelli, Chiara Bedin, Donato Nitti, Patrizia Pontisso

**Affiliations:** ^1^ Department of Medicine, University of Padua, Italy; ^2^ Surgery Branch, Department of Surgery Oncology and Gastroenterology, University of Padua, Italy; ^3^ Nano-Inspired Biomedicine Lab, Istituto di Ricerca Pediatrica - Città della Speranza, Padua, Italy; ^4^ Department of Nanomedicine, The Methodist Hospital Research Institute, Houston, TX, USA

**Keywords:** colorectal cancer, SerpinB3, COX-2, β-Catenin, tumor stage

## Abstract

Colorectal cancer is characterized by aberrant Cyclooxigenase-2 (COX-2) and β-Catenin pathways. Recently, the protease inhibitor SerpinB3 has been described overexpressed in more advanced stages of this tumor. Aim of the study was to explore the possible relationship between these molecules in this setting. We evaluated colorectal cancer specimens from 105 patients and a positive correlation between SerpinB3, COX-2 and β-Catenin expression was observed, with higher levels in tumor than in adjacent tissue. The highest levels were associated with pathologic parameters of poor prognosis, including vascular invasion, lymph node metastasis and perineural invasion. The molecular and protein profiles of COX-2 and β-Catenin were analyzed in cell lines with different expression of SerpinB3. In those with high expression of SerpinB3, COX-2 and β-Catenin were higher than in controls. Cells with high levels of SerpinB3 showed higher proliferation and invasion compared to controls. In conclusion, in colorectal cancer SerpinB3, COX-2 and β-Catenin are positively correlated and associated with more advanced tumor stage. The *in vitro* experimental results support a driving role of SerpinB3 in the upregulation of COX-2/ β-Catenin positive loop, associated with a more aggressive cellular phenotype.

## INTRODUCTION

Colorectal cancer (CRC) is a multistep process characterized by genetic aberrations of several oncogenes. In particular, many studies have demonstrated that *K-RAS* mutations occur in 30% to 50% of colorectal tumors [1–7]. Recently, Catanzaro et al. have demonstrated that mutant K-RAS induces inflammatory cytokine production and tumorigenesis by upregulating SerpinB3 and SerpinB4 isoforms, both members of the serine/cysteine protease inhibitors family [8]. These serpins have been previously reported to stimulate liver carcinogenesis [9]. Indeed, their over-expression promotes cell survival through the inhibition of apoptosis [10, 11], increases cell proliferation and induces epithelial-mesenchymal transition (EMT) [12, 13]. In liver cancer with poor prognosis SerpinB3 was associated with TGF-β1 and β-Catenin expression [14]. One of the major pathways involved in EMT regulation is the Wnt/β-Catenin pathway [15, 16] which is also one of the most important pathways associated with colorectal cancer progression and it was shown to be constitutively activated in about 90% of sporadic colorectal tumors [17–19]. In particular, β-Catenin induces the over-expression of cyclooxygenase-2 (COX-2 or PTGS2). COX-2 enhances prostaglandin E2 (PGE_2_) levels, can induce inflammation, cell proliferation, angiogenesis, EMT and invasiveness and it was found increased in more advanced forms of breast, colon, biliary tract, skin, lung and liver cancer [20–23]. COX-2/PGE2 may exert their pro-oncogenic action through the stimulation of the β-Catenin/LEF1/TCF-mediated transcription, which is known to activate colorectal carcinogenesis [24]. In addition, recent studies have shown that β-Catenin and COX-2 are often co-expressed in cancer cells and that the latter is up-regulated by nuclear β-Catenin accumulation [25–29].

Aim of the present study was to clarify the relationship between SerpinB3, COX-2 and β-Catenin in colorectal cancer analyzing tumor samples of well characterized patients and cell lines with different expression of SerpinB3.

## RESULTS

### SerpinB3, COX-2 and β-Catenin mRNA expression in colorectal cancer specimens

**Figure 1:**
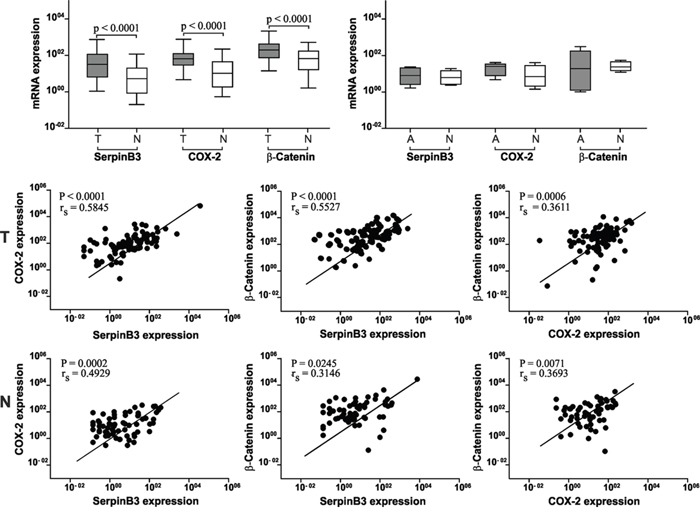
SerpinB3, COX-2 and β-Catenin mRNA expression levels in colorectal cancer tissue Upper panel: Expression levels of SerpinB3, COX-2 and β-Catenin are significantly higher in tumor (T) samples than in the corresponding non-tumor tissue (N). In adenoma (A) samples the levels of the three molecules are similar to the adjacent tissue (N). Results are expressed as pg/μL. Boxes and whiskers represent the 25th to 75th and 5th to 95th percentiles, respectively; the median value is represented by the central line in each box and the range of values of all samples is represented by vertical bars. Statistical analysis was carried out using Mann Whitney test. Lower panels: Correlation of mRNA levels between SerpinB3, COX-2 and β-Catenin mRNA in tumor (T) specimens and in the corresponding non tumor samples (N). r_s_ = Sperman correlation coefficient.

The absolute quantification of SerpinB3, COX-2 and β-Catenin mRNA expression, normalized by the HPRT1 housekeeping gene, was assessed in 105 primary human colorectal tumors (T) and in their corresponding adjacent non-tumor mucosa (N) by quantitative Real-Time PCR. A significant higher expression of all three genes was detected in tumor tissue, compared to matched non-tumor tissue (Figure 1, upper panel). In line with these results, a significant correlation was found between the expression of the three molecules that was higher in tumor specimens, especially between COX-2 and SerpinB3 (Figure 1, lower panels). In the adenomas transcript levels of the three genes were similar to those found in the colonic adjacent mucosa (Figure 1, upper panel). Despite the limited number of cases, it is worth to note that SerpinB3 values progressively increased from patient with low to high grade displasia, while the behaviour of COX-2 and β-Catenin were not related with the degree of displasia (Supplementary Table 1).

### Correlation of SerpinB3 mRNA with pathological data

**Figure 2:**
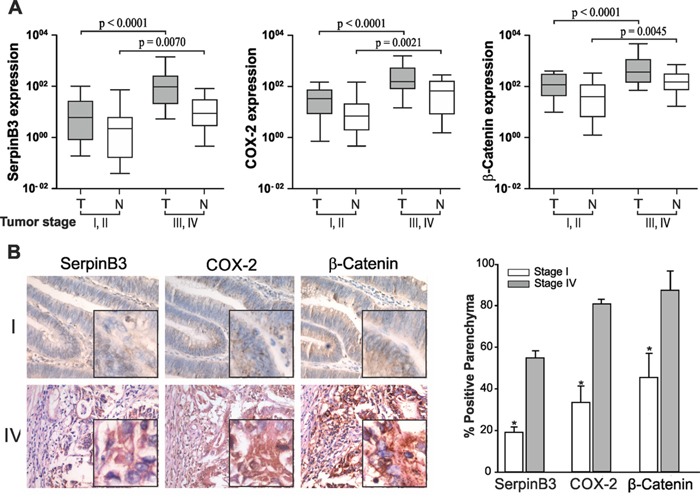
Correlation between SerpinB3, COX-2, β-Catenin expression and tumor stage **A**. Quantitative Real-Time PCR analysis of SerpinB3, COX-2 and β-Catenin expression levels in different tumor stages. Tumor (T) and non tumor (N) samples of early (I and II) and advanced (III and IV) tumor stages were analyzed using the Mann-Whitney test. Boxes and whiskers represent 25th to 75th and 5th to 95th percentiles, respectively; the median value is represented by the central line in each box and the range of values of all samples is represented by vertical bars. **B**. Immunohistochemical staining for SerpinB3, COX-2 and β-Catenin obtained in serial sections of a stage I and of a stage IV samples. In stage IV nuclear and cytoplasm positivity of SerpinB3 protein was detectable in a large number of cells, while in stage I only a small number of cells showed low level of expression. COX-2 was strongly positive in the cytoplasm and in the perinuclear area of stage IV tumor and the highest reactivity was found at the bottom of the crypts, decreasing along the longitudinal axis to the luminal region. A similar result was obtained for β-Catenin. Magnifications: 200 X, inserts 400X. In the right panel, graphical representation of the quantitative analysis of each staining has been provided. The average sum of intensities and stained area percentage of each patient was calculated using JmageJ software. Values are the mean ± SD (bar) of 10 different images analyzed [*p<0.001, Low vs High].

In order to further elucidate the potential involvement of SerpinB3 in colorectal tumor progression, we analyzed its expression levels in relation to disease stage. A significant increase in SerpinB3 expression levels was found in more advanced stages (III-IV), both in tumor and non-tumor samples, although absolute values were higher in tumor specimens (Figure 2A). COX-2 and β-Catenin showed similar expression profiles. LabChip^®^ Systems results confirmed quantitative data, as shown in Supplementary Figure 1.

Immunohistochemistry results were in line with transcription data (Figure 2B) and the highest protein expression of all three molecules was found in stage IV tumors, while trivial levels were observed in stage I tumors, as reported in Figure 2B. It is worth to note that in sequential tumor sections the highest signal of SerpinB3 was detected in areas showing high positivity for COX-2 and β-Catenin, indicating a possible relationship among these molecules. In sections of non-tumor mucosa SerpinB3 was barely detected and a faint positivity of COX-2 and β-Catenin was observed mainly in the cytoplasm (Supplementary Figure 2).

**Figure 3:**
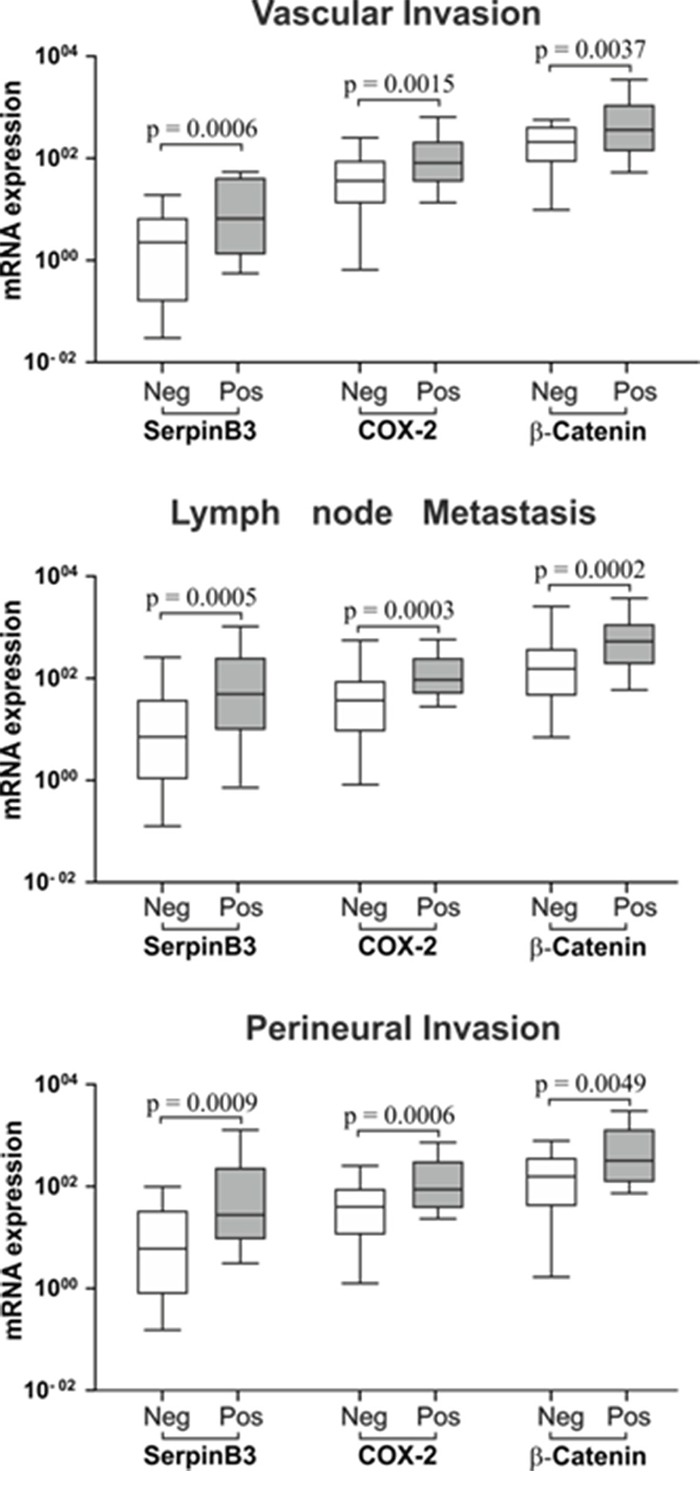
SerpinB3, COX-2 and β-Catenin expression in tumor specimens in relation with histological parameters of poor prognosis Mann-Whitney Test analysis was applied to determine statistical differences between positive versus negative groups for Vascular Invasion, lymph node metastasis and perineural invasion. Boxes and whiskers represent the 25th to 75th and 5th to 95th percentiles, respectively. The central line in each box represents the median value, the range of values of all samples is represented by vertical bar. The level of significance was set at p < 0.05. Neg = negative and Pos = positive for the pathologic parameter.

To assess the prognostic value of SerpinB3, COX-2 and β-Catenin, their expression in tumor specimens was analyzed in relation to pathologic parameters of poor prognosis, including vascular invasion, lymph node metastasis and perineural invasion. Tumor specimens positive for these pathologic parameters had significantly higher expression of SerpinB3, COX-2 and β-Catenin, compared to negative ones (Figure 3). In the corresponding non tumoral tissue the expression of SerpinB3 and COX-2, but not that of β-Catenin, was significantly higher in patients with lymph node metastasis, while vascular and perineural invasion did not affect the expression of these molecules (Supplementary Figure 3).

### Expression in cell cultures

**Figure 4:**
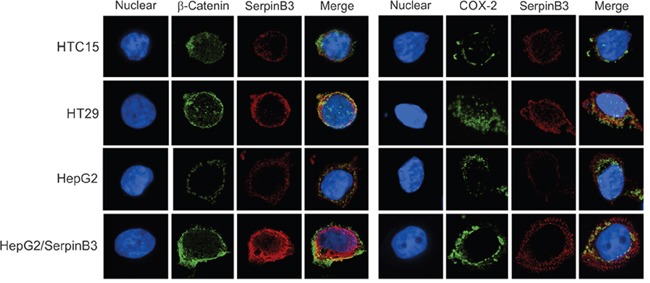
Immunofluorescence analysis of SerpinB3, COX-2 and β-Catenin Immunofluorescence staining of HTC15, HT29, HepG2 and HepG2/SerpinB3 cell lines after 48 hour of culture. Left panel: Double staining for SerpinB3 and β-Catenin proteins. Right panel: Double staining for SerpinB3 and COX-2 proteins. Proteins were visualized under a fluorescent microscope: TRIC (red, SerpinB3), FITC (green, β-Catenin and COX-2) and DAPI (blue, nuclei). Original magnification 400 X

In preliminary experiments we have analyzed SerpinB3 expression in different human colon carcinoma cell lines (RKO-E6, SW48, HCT 116, HT29 and HTC 15). HT29 cells showed a strong SerpinB3 expression signal, while in the other cell lines only a weak positivity was observed. For this reason, we have chosen HT29 as positive colon cell line and HTC15 as negative control cell line. The previously studied HepG2/SerpinB3 cell line that stably expresses SerpinB3 [12] was used as further positive control and HepG3 cells transfected with the plasmid vector alone were used as additional negative control (HepG3/Control). Fluorescence and ROI data confirmed the correlation of this serpin with the extent of β-Catenin and COX-2 expression (Figure 4 and Supplementary Figure 4).

**Figure 5:**
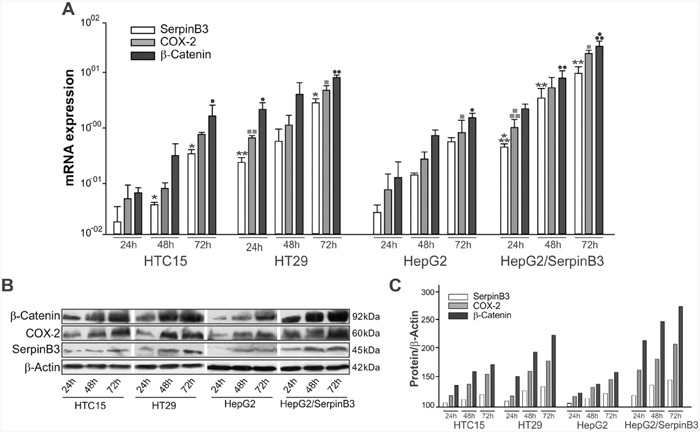
Profile of expression of SerpinB3, COX-2 and β-Catenin in cell lines **A**. In HTC15, HT29, HepG2 and HepG2/SerpinB3 cell lines SerpinB3, COX-2, and β-Catenin transcripts were quantified at 24, 48 and 72 hours after plating by real-time PCR. Results are expressed as mean +/− SE of three independent experiments. SerpinB3: HTC15 cell line 24hr versus 72hr *p = 0.0376 and 48hr versus 72hr *p = 0.0376; HT29 cell line 24hr versus 48hr **p = 0.0018, 24hr versus 72hr *p = 0.0181 and 48hr versus 72hr *p = 0.0134; HepG2/SerpinB3 cell line 24hr versus 48hr ***p < 0.0001, 24hr versus 72hr **p = 0.0014 and 48hr versus 72hr **p = 0.0028. COX-2: HT29 cell line 24hr versus 48hr ▪▪p = 0.0098, 24h versus 72h ▪p = 0.0316 and 48h versus 72h ▪p = 0.0103; HepG2 cell line 24h versus 72hr ▪p = 0.0180; HepG2/SerpinB3 cell line 24hr versus 48hr ▪▪▪p = 0.0002, 24hr versus 72hr ▪▪p = 0.0023. β-Catenin: HTC15 cell line 24hr versus 72hr ·p = 0.0421; HT29 cell line 24hr versus 48hr ··p = 0.0047, 24hr versus 72hr ··p = 0.0065 and 48hr versus 72hr ·p = 0.0201; HepG2 cell line 24hr versus 72hr ·p = 0.0300; HepG2/SerpinB3 cell line 24hr versus 48hr ···p = 0.0004 and 48hr versus 72hr ··p = 0.0024. **B**. SerpinB3 (45 KDa), COX-2 (60 KDa) and β-Catenin (92 KDa) proteins were analyzed by Western blot and **C**. Their levels were estimated by densitometric analysis (QuantityOne software, Biorad). β-Actin expression was used for sample normalization. Results are representative of three independent experiments.

Time course analysis revealed that COX-2 and β-Catenin genes were over-expressed at transcription and protein level in cells with high levels of SerpinB3 (Figure 5), while lower levels of these molecules were found in cells with low SerpinB3. HT29 and HepG2/SerpinB3 cell lines showed indeed similar profiles, as occurred for HTC15 and HepG2/Control cell lines.

HT29 cells, expressing high levels of SerpinB3, showed higher proliferation rate and invasion ability, compared with HTC15 cells (Supplementary Figure 5). The effect of the exposure to recombinant SerpinB3 was also assessed in HTC15 and HT29 cell lines. A significant increase of proliferation, but not of invasion, was observed in both cell lines exposed to SerpinB3 and the result was more prominent in the HT29 cell line (Supplementary Figure 5). These findings recall previous data obtained in HepG2 cells, however, in the hepatoma cell line exogenous SerpinB3 determined also a significant increase of invasion [12].

The exposure to recombinant SerpinB3 did not determine any significant increase of β–Catenin, mainly detected in the cytosolic fractions, and of COX-2, mainly detected in the nuclei (Supplementary Figure 6). It is worth to note that this exocrine stimulation determined also some increase of nuclear SerpinB3, especially in the HT29 cell line. This nuclear form of the serpin showed higher molecular weight, compared with the cytosolic counterpart, suggesting its binding to another, not yet characterized, molecule and this finding deserves further studies.

## DISCUSSION

Several studies have shown the important role of COX-2 and β-Catenin proteins in colorectal tumorigenesis. Recently, SerpinB3/4 isoforms have been found upregulated by oncogenic Ras in advanced stages of colorectal and pancreatic tumors [8]. To our experience, this is the first study in which SerpinB3 has been analyzed in colorectal cancer and correlated with the expression of COX-2 and β-Catenin. Previous observations indicate that COX-2 and β-Catenin expression was higher in poorly differentiated stages than in well or moderately differentiated stages [30–32]. Our results have demonstrated that SerpinB3, COX-2 and β-Catenin were significantly higher in tumor tissues, compared to the normal counterpart and that their expression progressively increased according to tumor stage. Moreover, tumor specimens with pathologic parameters of poor prognosis, including vascular invasion, lymph node metastasis and perineural invasion showed significantly higher expression of all these three molecules.

*In vitro* results suggest that SerpinB3 increases cell proliferation and invasion and that this serpin is the driver of COX-2 and β-Catenin behaviour. Their expression was indeed dependent on the presence of this serpin. It should be noted that the HT-29 cell line, overexpressing SerpinB3, shows enrichment of cancer stem cell markers [33, 34] and this could be one of the possible explanations for its increased aggressiveness and/or its increased expression of SerpinB3. These results are in agreement with our previous study carried out in hepatocellular carcinomas, where high expression of SerpinB3 in neoplastic livers was characterized by high β-Catenin levels and more disseminative clinical phenotype, evidenced as more frequent early tumour recurrence [14]. In addition, SerpinB3 has been found expressed also in hepatic progenitor cells and it is well known that tumors showing hepatic progenitor cell features have a worse prognosis and a higher recurrence rate compared to tumors lacking these characteristics [35].

**Figure 6:**
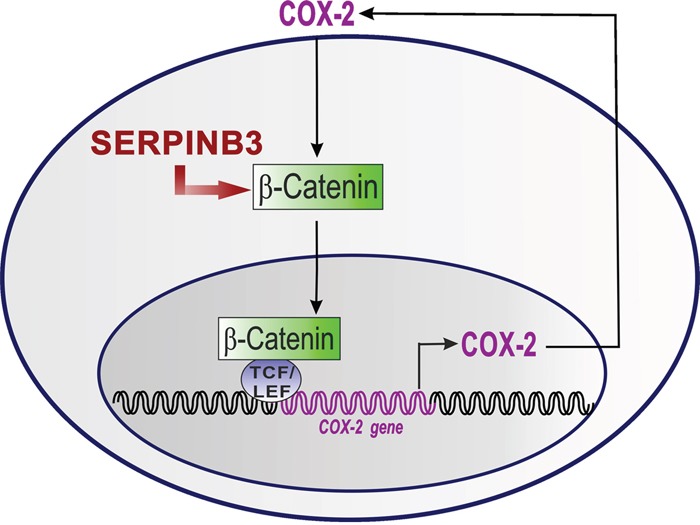
The proposed mechanism the COX-2/ β-Catenin positive loop upregulation by SerpinB3 SerpinB3 determines an increase of β-Catenin. The accumulation of β-Catenin in the nucleus interacts with T-cell factor (TCF) and lymphoid enhancer binding protein (LEF) transcription factors to activate COX-2 gene. The COX-2 protein in turn increases PGE_2_ levels and these two molecules strengthen the positive loop of β-Catenin/LEF1/TCF-mediated transcription.

On the basis of the results obtained in our study, we could hypothesize that SerpinB3 may be involved in the induction of β-Catenin accumulation that promotes the activation of COX-2/PGE2 axis which, in turn, contributes to maintain their positive loop (Figure 6). The best proof of concept of this novel nexus could be supported by the use of a SerpinB3 inhibitor, but at best of our knowledge, no inhibitors of this serpin have been reported yet in the literature. In addition, we cannot exclude that SerpinB3 may lead COX-2 induction also through the activation of NF-kB dependent inflammatory cascade [36], as a result of the block of protein turnover determined by this serpin.

In conclusion, in patients with colorectal cancer the expression of SerpinB3 was higher in more advanced tumor stages and it was correlated with histological parameters of poor prognosis. This serpin was shown to induce the expression of β-Catenin and of COX-2, molecules already identified as markers of poor prognosis, and it might be considered as a novel molecular target suitable for colorectal cancer therapy.

## MATERIALS AND METHODS

### Patient Characteristics

Surgical samples were obtained from 105 patients who underwent surgery for colorectal cancer as first line therapy at the Surgical Clinic I of the Padua University Hospital between 2002 and 2005. Table 1 summarizes clinical and histopathological features of patients included in the study. Tumor and the adjacent non tumor tissue samples were collected at the time of surgical resection and part was formalin fixed and paraffin embedded, while the remaining part was immediately shock-frozen at -80°C for further analysis.

**Table 1:** Clinical and histopathological characteristics of the patients included in the study

PATIENT CHARACTERISTIC	n.	(%)
**Gender**		
Female	45	(42.9)
Male	60	(57.1)
**Age**		
≤ 60 years	24	(22.9)
> 60 years	81	(77.1)
Median (years)	66	
Range (years)	32-87	
**Histology**		
**Risk**		
Sporadic	34	(32.19
Familiarity	5	(4.7)
S-HNPCC	4	(4.7)
Unknown	62	(58.0)
**Tumor Type**		
Adenoma	5	(4.8)
Adeno-carcinoma	92	(87.6)
Mucoid-carcinoma	8	(7.6)
**Tumor Site**		
Right colon	33	(31.4)
Left colon	61	(58.1)
Rectal	11	(10.5)
**Histological Grade**		
Well differentiated (1)	15	(14.2)
Moderately (2)	67	(63.2)
Poorly (3-4)	12	(11.3)
Unknown	11	(10.5)
**pTNM Stage**		
I	32	(30.2)
II	27	(24.5)
III	16	(15.09)
IV	25	(23.58)
Unknown	5	(4.8)
**Vascular Invasion**		
Positive	48	(45.7)
Negative	49	(46.6)
Unknown	8	(7.6)
**Lymph nodes Metastasis**		
Positive (N1+2)	32	(30.5)
Negative (N0)	65	(61.9)
Unknown	8	(7.6)
**Perineural Invasion**		
Positive	24	(22.8)
Negative	72	(68.6)
Unknown	9	(8.6)

S-HNPCC= Syndrome-hereditary non-polyposis colorectal cancer. N1+2= metastasis in more regional lymph nodes. N0= no regional lymph node metastasis.

Pathological samples were classified according to the AJCC staging classification, using the pTNM system [37]. The study was approved by the local Ethical Committee (Ethical Committee of the Padua Teaching Hospital, Approved Protocol Number: P.448) and all patients providing written informed consent were prospectively registered in the First Surgical Clinic Tissue Biobank database at the Padua Hospital, according to national and international guidelines.

### Cell lines and culture conditions

Two colon cell lines with different extent of SerpinB3 expression were preliminary selected: the HTC15 (ATCC CCL-225) and the more undifferentiated HT29 (ATCC HTB-38) cell lines [38–40]. In some experiments recombinant SerpinB3 (rSerpinB3) was also used overnight at 200ng/ml concentration to assess its paracrine effect on these cell lines. As positive control, the HepG2 cell line (ATCC-HB-8065) stably transfected to overexpress SerpinB3 [12] was used, while HepG2 cells transfected with the plasmid alone and expressing trivial levels of SerpinB3 were used as negative control (HepG2/Control). The HepG2 transfected cells are regularly tested for SerpinB3 expression by RT-PRC and by immunofluorescence at each thawing of new frozen vials. HTC15 cells were cultured in RPMI 1640 medium (ThermoFisher Scientific, Milan, Italy) and HT29 cells were cultured in McCoy's Modified Medium (ThermoFisher Scientific) either supplemented with 10% heat-inactivated fetal calf serum (FCS, ThermoFisher Scientific), 20 mM *L-*glutamine (Sigma Aldrich, Milano, Italy), and 100 U/mL streptomycin. HepG2/Control and HepG2/SerpinB3 cells were maintained in standard medium supplemented with 1 μg/mL Geneticin (G418; Sigma Aldrich, Milano, Italy) as previously described [12]. Each cell line was plated with a density of 400.000 cells/well, maintained in a humidified 5% CO_2_ incubator at 37°C and after 24, 48 and 72 hour incubation cells lines were harvested and washed with 1× PBS (137 mM NaCl, 2.7 mM KCl, 10 mM Na_2_ HPO_4_, 2 mM KH_2_ PO_4_, pH 7.2).

The real time cell analyzer (RTCA, xCELLigence, ACEA Biosciences) was used for real-time cell proliferation and invasion analysis. Briefly, cellular proliferation was measured as impedance-based signals, reflecting cell adherence. To measure cellular invasion cells were plated on top of an upper chamber equipped with 8-μm porosity filter coated with Matrigel 1:20 in basal medium (BD, Biosciences). As cells moved towards the lower chamber, containing fetal bovine serum as chemo-attractant, they adhered to gold impedance microelectrodes, determining a change in the impedance signal. The Cell Index, reflecting the cell number present in well, was monitored every 10 minutes and calculated as the relative change of electrical impedance caused by migrated cells using the RTC software (version 1.2, Roche Diagnostics). All the results were expressed as mean of experiments performed in quadruplicate.

### RNA isolation and real-time PCR

Extraction of total cellular RNA and reverse transcription were carried out according to a previously published protocol [41]. Total RNA was reverse transcribed using SuperScript III reverse transcriptase (Invitrogen) for 3hr at 55 °C. For SerpinB3 and β-Catenin genes specific sense and antisense primers were used as previously described [15], while for COX-2 the following primers were used: forward 5′ TCA AAT GAG ATT GTG GAA AAA T'3 and reverse 5′ AGA TCA TCT CTG CCT GA GTA TCT T '3. To quantify the amount of mRNA in each sample, a standard reference curve was realized using serial 5-fold dilutions of HT29 cDNA (from 10 to 0.003 pg/reaction). Hypoxanthine guanine phosphoribosyl transferase 1 (HPRT1) did not show statistically significant difference between tumor and non-tumor mucosa and it was chosen as housekeeping gene. Each sample was run in duplicate and expression data were normalized to 10^3^ copies of HPRT1. The results were reported as ng of expressed mRNA [42].

In some representative cases, quantitative results obtained by Real-Time PCR were confirmed by the LabChip® Systems (LabChip® GX Touch for Genomics, PerkinElmer, Milan, Italy), according to the manufacturer's instructions.

### Protein expression

The expression of each protein was detected by Western blot using the following monoclonal primary antibodies against: SerpinB3/SCCA1 (clone 8H11, 2μg/mL Santa Cruz Biotechnology, Heidelberg, Germany; Hepa-Ab, 8 μg/mL, Xeptagen, Venice, Italy), COX-2 (clone MTC02, 2 μg/mL, Santa Cruz Biotechnology, Heidelberg, Germany), β-Catenin (clone 17C2, 0.4 μg/mL, Novacastra, Leica Biosystems, Newcastle, UK) and β-actin (clone AC15, 0.018–10 μg/mL, Sigma Aldrich, Milan, Italy). Nuclear and cytosolic extracts were prepared by lysing HTC15 and HT29 cells in ice with isolation buffer (250 mM sucrose, 10mM tris-HCl, o,1 mM EGTA, pH7,4) including phosphatase and protease inhibitors. Cells were mechanically homogenized with pestles and centrifuged at 900g for 10 minutes. Pellet containing nuclei was resuspended in RIPA lysys buffer (Millipore) and supernatant containing cytoplasm was added with 0,5% Triton X-100. For immunoblotting analysis 30 ug of total lysates or of cellular fractions cells were loaded on 10% SDS-polyacrylamide gels and proteins were blotted on nitrocellulose membranes following standard methods. Nuclear matrix protein P84 and GAPDH were used to normalize protein extracts expression. After incubation with the different antibodies, the results were visualized by the enhanced chemiluminescence method using standard protocols (Pierce Biotechnology, Rockford, USA).

Immunohistochemistry was carried out on 3-μm thick paraffin sections of tumor and non tumor samples of 16 patients. Images of representative fields were captured by Leica Qwin Plus v3 software, under a CCD camera connected to a Leica M165FC microscope (Microsystem Imaging Solutions Ltd. Milan, Italy). The immunoreactivity was semi-quantified using the x JmageJ software (https://imagej.nih.gov/). Positivity was expressed as percentage of positive areas over total area. Data represent the average of all the 6 fields acquired for each analyzed section. Staining specificity was confirmed by replacing primary antibody with PBS.

For immunofluorescence analysis cells were seeded on slides (5×10^5^ cells/well) in a maximum volume of 3 mL/well, cultured for 2 days and then fixed in 4% paraformaldehyde. Primary antibodies described above were used, followed by appropriate secondary antibodies at room temperature for 60 min, TRITC-conjugated anti-rabbit antibody and FITC-conjugated anti-mouse antibody (1 μg/mL; Dako, Copenhagen, Denmark). Nuclei were stained with Hoechst 33342 (20 μg/mL; Sigma Aldrich, Milan, Italy). Slides were analyzed using a video-confocal fluorescence microscope ViCo C2 (Nikon, Florence, Italy) equipped with a triple band pass filter set (FITC, TRITC, DAPI). Mean fluorescence intensity in regions of interest (ROI) was measured by using the ImageJ/Fiji ROI Manager.

### Statistical analysis

All data were presented as mean±standard error (SE) of the mean and were analyzed by Mann–Whitney U test, Spearman rank correlation test, when appropriated, using GraphPad Prism version 5.0 for Windows (GraphPad Software, San Diego, CA) and Social Sciences (SPSS, version 20.0, Chicago, USA). A *P* value < 0.05 was considered statistically significant. Student's t-test was applied to determine statistical significance between different time points ( 24hr versus 48hr - 72hr and 48hr versus 72hr) in the four cell lines. All statistical tests were two-sided (see Supplementary Material and Methods).

## SUPPLEMENTARY MATERIALS FIGURES AND TABLES


