# Supplementary material for: SerpinB3 upregulates the Cyclooxygenase-2 / β-Catenin positive loop in colorectal cancer
Source: Oncotarget. 2017 Feb 2;8(9):15732–43. doi: 10.18632/oncotarget.14997 (PMC5362519; doi:10.18632/oncotarget.14997)
Supplement: Supplementary file 1 [file oncotarget-08-15732-s001.pdf]

SerpinB3 upregulates the Cyclooxygenase-2 / β-Catenin positive loop in colorectal cancer

SUPPLEMENTARY DATA

SUPPLEMENTARY MATERIALS AND METHODS

Patient characteristics

This retrospective analysis included biopsy samples obtained from 105 patients (60 male and 45 female) with a median age of 66 years (range 32-87 years), with a diagnosis of CRC and who underwent surgery at a single Institution from 2002 to 2005. Ninety-seven patients with colon cancer (92 with colonic carcinomas of which 26 right colon and 55 left colon, 11 with rectal carcinomas and 5 with colonic adenomas of which 3 right colon and 2 left colon), 7 patients with mucinous adenocarcinomas (MUCs) (3 right colon and 4 left colon) and 1 patient non valuable histologicaly, who were admitted to the Department of Surgical Sciences, Oncology and Gastroenterological, Surgical Clinic Section I Hospital (Padua, Italy). Sixty-four adjacent non-tumors tissue samples (N) with normal histology were available for this study. All of whom had an adenocarcinoma classified following the tumour node metastasis (TNM) staging system: 26 patients with stage IV (any T1, any N, M0 M1) tumors [median age: 61 (range 32-80) years; 14 males and 11 females; tumour location: right colon n = 9, left colon n = 13 and rectum n = 4] of which 15 with vascular invasion, 17 with lymph node metastatic/total and 11 with perineural invasion; 16 patients with stage III (any T, N1-N2, M0) tumors [median age: 68 (range 51-87) years; 9 males and 7 females; tumour location: right colon n = 4 and left colon n = 12] of which 11 with vascular invasion, 14 with lymph node metastatic/total and 5 with perineural invasion; 26 patients with stage II (T3-T4 N0 M0) tumors [median age: 69 (range 51-86) years; 14 males and 13 females; tumour location: right colon n = 7, left colon n = 16 and rectum n = 3] of which 13 with vascular invasion, 1 with lymph node metastatic/total and 7 with perineural invasion; 32 patients with stage I (T1-T2 N0 M0) tumors [median age: 66 (range 45-83) years; 20 males and 12 females; tumor location: right colon n = 9, left colon n = 19 and rectum n = 4] of which 8 with vascular invasion, no lymph node metastatic/total and 1 with perineural invasion. Finally, 5 patients with stage 0. Table 1 summarizes the clinical and histopathological findings in our CRC patients.

Tissue processing and analysis

For all patients, tissues were obtained at the time of surgery and none of the patients underwent radiotherapy or chemotherapy before surgery. Samples from both the cancer lesion and the adjacent non-tumor tissue were obtained for each CRC patient and were immediately snap-frozen in liquid nitrogen and stored at -80°C until use.

The study was approved by the local Ethics Committee and all patients were prospectively registered in a central secured database according to the Commission National AJCC/UICC Classification. Written informed consent was obtained from all subjects.

Cell lines and culture conditions

In this study the following cell lines were analysed: the Dukes' type C colorectal adenocarcinoma HTC15, the colorectal adenocarcinoma HT29, the hepatoma cell line HepG2 and the HepG2/SerpinB3 cell clone, expressing high levels of SerpinB3 (Quarta S. et al., 2010).

HT29 cells were cultured in McCoy’s Modified Medium (Invitrogen, Life Technologies, Monza, Italy) supplemented with 10% heat-inactivated fetal calf serum (FCS, Invitrogen, Life Technologies, Monza, Italy), 100 U/mL penicillin, 100 μg/mL streptomycin, and 20 mM L-glutamine (Sigma Aldrich, Milano, Italy); the HTC15 cells were cultured in RPMI 1640 medium supplemented with 10% FCS and 2% glutamine. HepG2 and HepG2/SerpinB3 were maintained in standard medium supplemented with 1 μg/mL Geneticin (G418; Sigma Aldrich, Milano, Italy) as described previously (Quarta S et al. 2010). All cells line were maintained in a humidified 5% CO_2 incubator at 37°C. After 24, 48 and 72 hour incubation, the cells lines were harvested and washed with 1× PBS (137 mM NaCl, 2.7 mM KCl, 10 mM Na_2 HPO_4 , 2 mM KH_2PO_4, pH 7.2).

RNA extraction and mRNA expression

mRNA extraction

Total RNA was extracted from the tissue fraction using 1 mL RNasy Trizol reagent (Invitrogen, Life Technologies, Monza, Italy) according to the manufacturer’s instructions and quantified

by spectrophotometry at 260nm (NanoDrop spectrophotometer ND-1000, Thermo Fisher Scientific, Wilmington, USA). The cDNA was synthesized using SuperScriptIII reverse transcriptase (Invitrogen, Life Technologies, Monza, Italy) according to the manufacturer's instructions for 3 hour at 55°C and 15 minutes at 72°C, diluted with deionised water to 100 ng/µL; and preserved at 4°C.

mRNA expression

All qPCR assays were performed on an Thermal Cycler CFX96 Real Time-PCR detection system (Bio-Rad, Hercules, CA, USA) using CFX Manager software (Bio-Rad, Hercules, CA, USA); 5 µL of 2x SsoFast EVA Green SuperMix (Bio-Rad, Hercules, CA, USA), 2.5 µL of diluted cDNA (25 ng) and 0.2 µL (200 nmol/L) each of gene-specific sense and antisense primers. SerpinB3 and β-Catenin primers were previously described (Turato et al , 2010 and 2114), whereas for COX-2 they were as follows: forward 5' TCA AAT GAG ATT GTG GAA AAA T'3 and reverse 5' AGA TCA TCT CTG CCT GA GTA TCT T '3. Final reaction volume was 10 µL. After an initial denaturation step of 2 min during which the well factor was measured, 45 cycles of 10 s at 95°C followed by 30 s at 60°C were performed (Turato C et al., 2010). To determine the amount of SerpinB3, COX-2, and β-Catenin in each sample, a standard reference curve was realized using serial 5-fold dilutions of the cDNA obtained from RNA extracted of the HT29 cell line and retrotranscribed as described above (equivalent to 100, 50, 10, 2, and 0.4 pg of total RNA). The Ct values obtained from each sample were then plotted against the calibration weight curve. Each sample was run in duplicate and expression data were normalized for 10^3 copies of the housekeeping hypoxanthine guanine phosphoribosyl transferase 1 (HPRT1) gene (Terrin L et al., 2008).

Protein expression

The expression of each protein was detected using the following anti-human monoclonal primary antibodies: SCCA1 (clone 8H11, 2µg/mL), COX-2 (clone MTC02, 2 µg/mL) (Santa Cruz Biotechnology, Heidelberg, Germany), β-Catenin (clone 17C2, 0.4 µg/mL; Novacastra, Leica Biosystems, Newcastle, UK) and β-actin (clone AC15, 0.018–10 µg/mL, Sigma Aldrich, Milan, Italy).

Immunoblotting

Total protein extracts were obtained as described (Terrin L et al., 2008). The protein content was estimated using the BCA method with the BCA protein assay kit (Pierce, Rockford, IL, USA). Proteins were fractionated using SDS-PAGE and transferred onto nitrocellulose membranes. The lane band positions were marked and the membrane was washed thoroughly with TBS and was blocked with 3% BSA in TBS overnight at 4°C. Specific proteins were visualized using the enhanced chemiluminescence detection SuperSignal kit according to the manufacturer's instructions (Pierce Biotechnology, Rockford, USA). β-actin Western blot analysis was performed as a control of equal sample loading.

Immunohistochemistry

Immunohistochemical analysis was carried out on 3-µm thick paraffin sections as described (Turato et al., 2014). Sixteen patients with CRC (age range, 41-78 years) were stained with hematoxilin and eosin for histopathologic analysis; all tumour samples analyzed contained > 80% tumour cells. After overnight incubation with the primary antibody at +4°C, immunostaining was performed using the EnVision+System-HRP (DAB) kit and 3,3′-diaminobenzidine tetrahydrochloride chromogen as substrate (DakoCytomation, Milan, Italy). Staining without primary antibody was used to validate the specificity of the secondary antiserum. Finally, the cells were lightly counterstained with Mayer's haematoxylin.

Immunofluorescence

The cells were seeded on slides at 5 10^5 cells/well in a maximum volume of 3 mL/well for 2 days and then fixed in 4% paraformaldehyde, permeabilized in 0.2% Triton X100 (Sigma Aldrich, Milan, Italy) and saturated with 5% BSA-5% Normal Goat Serum/PBS (Vector Labs, Peterborough, UK) for 30 min at room temperature. Slides were then incubated overnight at 4°C with the primary antibodies against COX-2, β-Catenin described above. For SerpinB3 detection, the anti-human SCCA1 oligoclonal antibody was used (Hepa-Ab, 8 µg/mL, Xeptagen, Venice, Italy). For negative controls, primary antibodies were omitted. Primary immunoreaction was detected after washing with 0.1% Tween 20 in PBS following incubation with the appropriate secondary reagent at room temperature for 60 min (1 µg/mL; TRITC-conjugated anti-rabbit antibody and FITC-conjugated anti-mouse antibody; Dako, Copenhagen, Denmark). The cellular nuclei were stained by incubation with Hoechst 33342 (20 µg/mL; Sigma Aldrich, Milan, Italy) at 37°C for 5 min. Slides were washed in PBS, mounted with Elvanol (Sigma Aldrich, Milan, Italy), and the results were analyzed using a fluorescence microscope (Nikon ViCo Video Confocal Microscope, Florence, Italy) equipped with a triple band pass filter set (FITC, TRITC, DAPI). Evaluation of nuclear hybridization signals was performed in triplicate by different investigators and the mean fluorescence intensity in a region of interest (ROI) was measured over time by using the ImageJ/Fiji ROI Manager.

Statistical analysis

Analysis was performed using GraphPad Prism version 5.0 for Windows (GraphPad Software, San Diego, CA) and Social Sciences (SPSS, version 20.0, Chicago, USA). For each set of experiments, values were reported as means ± SD. For selected experiments, the results were reported as box plots showing the median, minimum and maximum values and 25-75th percentiles and were calculated at two-sided 95% confidence intervals (95% CI) and *p* value. The parametric Mann–Whitney U test was used to compare quantitative SerpinB3, COX-2 and β-Catenin variables and tumor stage. The Spearman rank correlation test was used to analyse the correlation between SerpinB3, COX-2 and β-Catenin expression. A *p* value of less than 0.05 was considered statistically significant. The univariate analysis Mann-Whitney test was also used to assess the association between SerpinB3, COX-2 and β-Catenin mRNA levels and clinical-pathologic features, such as pathologic tumor-nodes-metastasis (TNM) stage (early, I/II; advanced, III/IV), histologic grading (well-differentiated, moderately differentiated, and poorly differentiated), perineural invasion, lymph node metastasis and vascular invasion. The prognostic value of SerpinB3, COX-2 and β-Catenin expression was evaluated by standard survival analysis, using the Kaplan-Meier method, considered from the date of diagnosis until the latest follow-up or death, and the significance of the survival distribution in each group was tested by means of a log rank test.

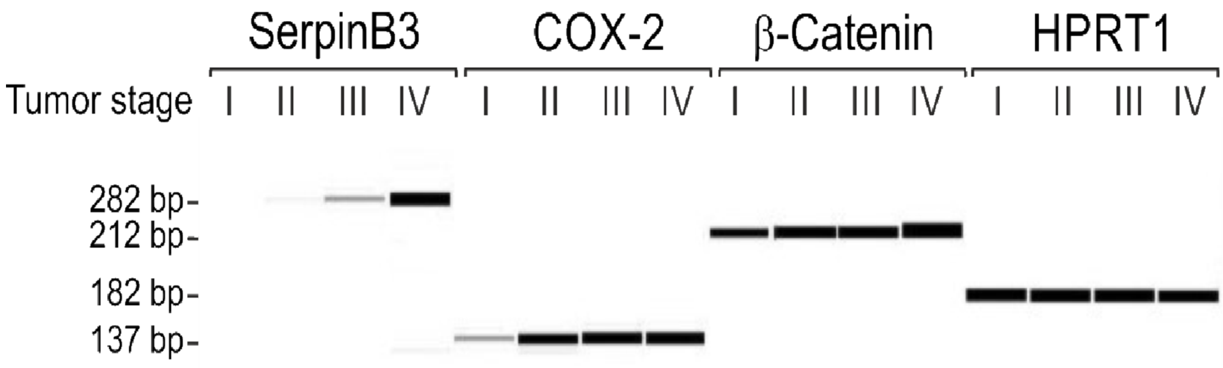

Supplementary Figure 1: Quantitative analysis of RNA expression. In representative tumor cases, positive or negative results obtained by Real-Time PCR were confirmed by LabChip® Systems technology.

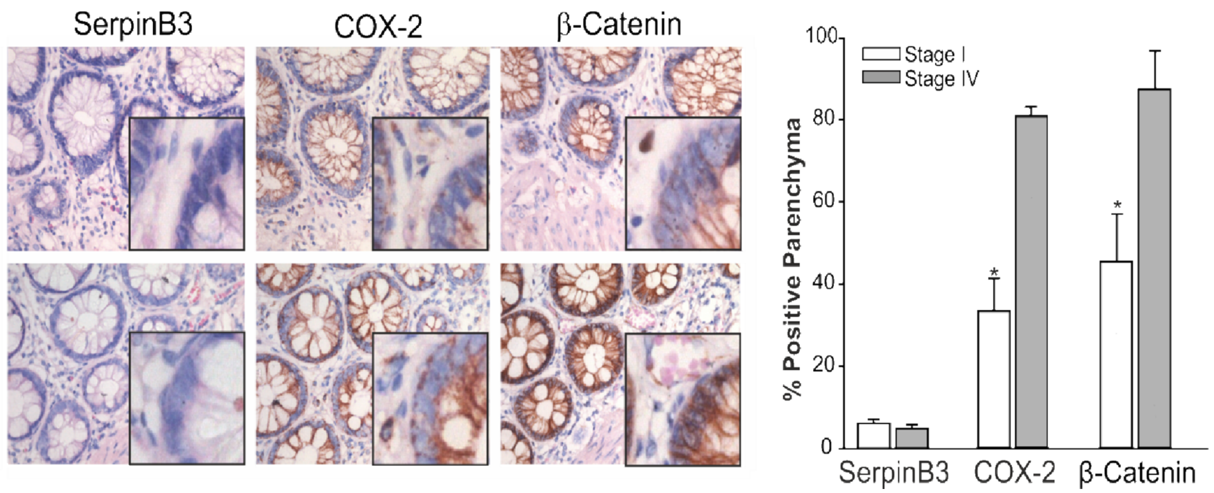

Supplementary Figure 2: SerpinB3, COX-2 and β-Catenin expression in non tumor samples. Representative examples of immunohistochemical staining for SerpinB3, COX-2 and β-Catenin were obtained in serial sections of a stage I non tumor and of a stage IV non tumor samples. In the section of non-tumor colon mucosa only the cytoplasm of a few epithelial cells and stromal cells were positively stained for COX-2 and β-Catenin. In the right panel, the bars represent the mean ± SD of 10 different images analyzed. The sum of intensities and stained area percentage of each patient was calculated using JmageJ software. Magnifications: 200 X, inserts 400 X [*p<0.001, Low vs High].

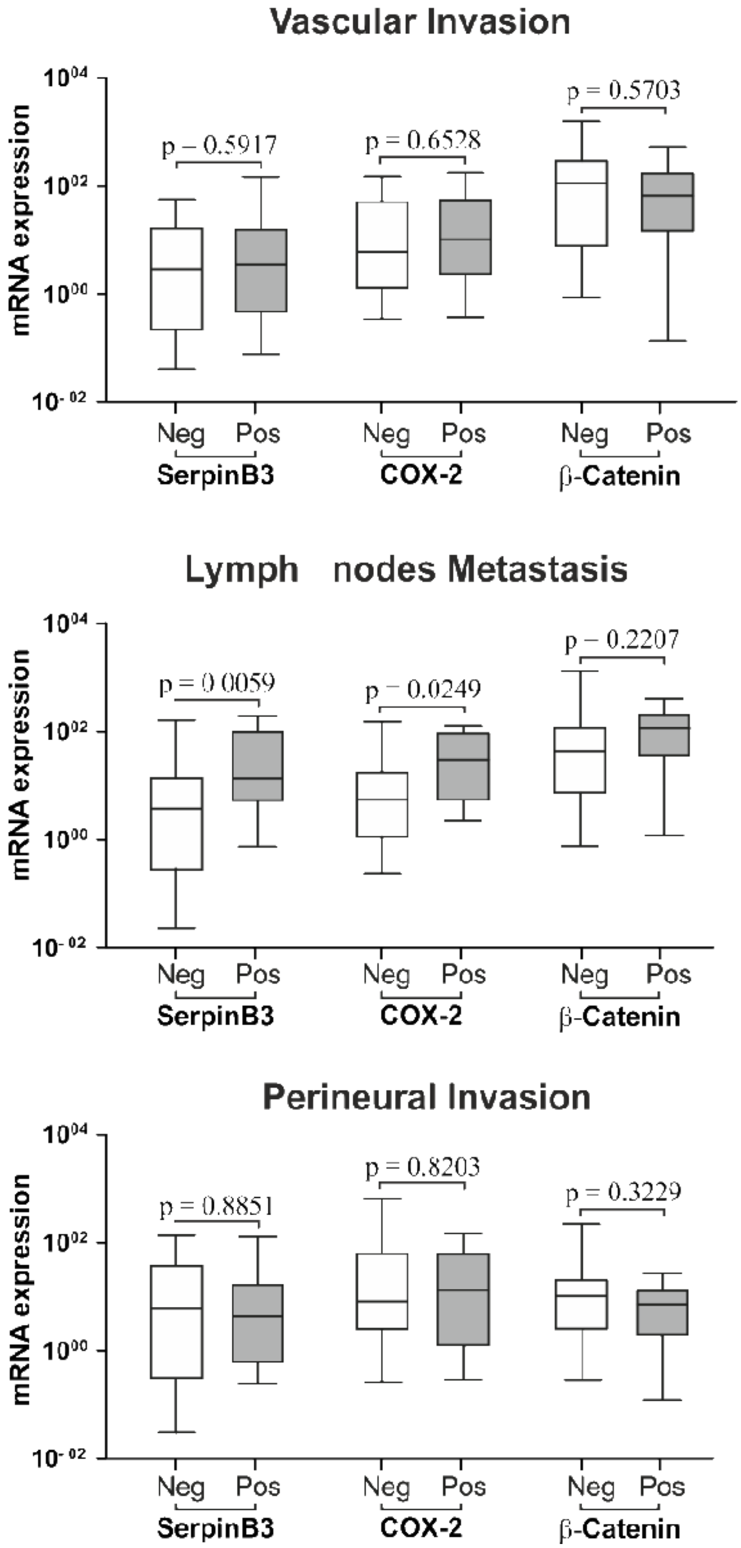

Supplementary Figure 3: SerpinB3, COX-2 and β-Catenin expression in non tumor specimens in relation with histological parameters of poor prognosis. Mann-Whitney test analysis was applied to determine the level of statistical significance for the differences between groups positive versus negative for Vascular Invasion, Lymph node metastasis and peri-neural invasion. Boxes and whiskers represent the 25th to 75th and 5th to 95th percentiles, respectively; the central line in each box represents the median value, the range of values of all samples is represented by vertical bar. Level of significance was set at $p < 0.05$. Neg = negative and Pos = positive.

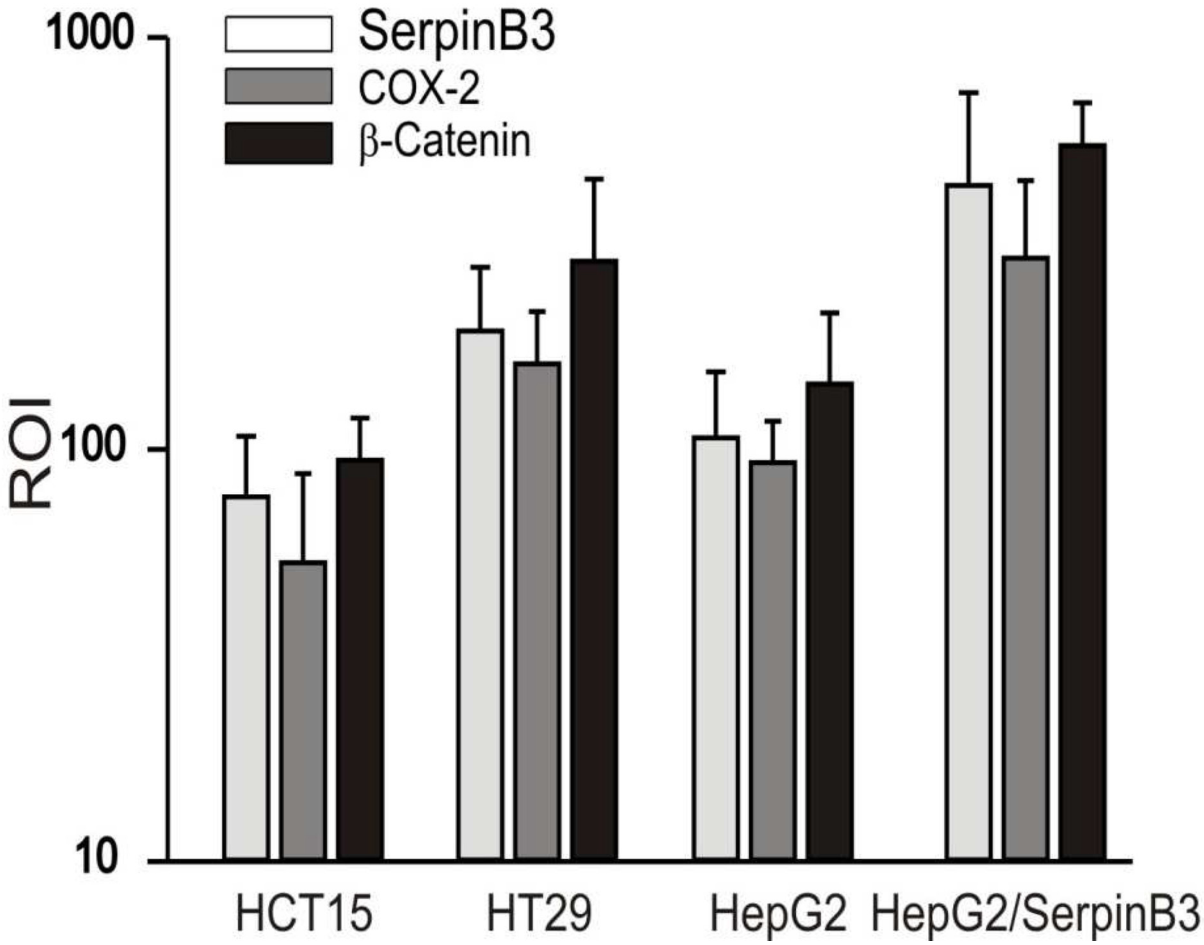

Supplementary Figure 4: Immunofluorescence quantification data. Immunofluorescence quantification of SerpinB3, COX-2 and β-Catenin in different cell lines was carried out using the ImageJ/Fiji ROI Manager software. Data are expressed as mean ± Standard Error of the percentage of positive staining in the region of interest (ROI) obtained in two independent experiments. Bar graphs show the total integrated signal per cell in the ROI, corrected against the background.

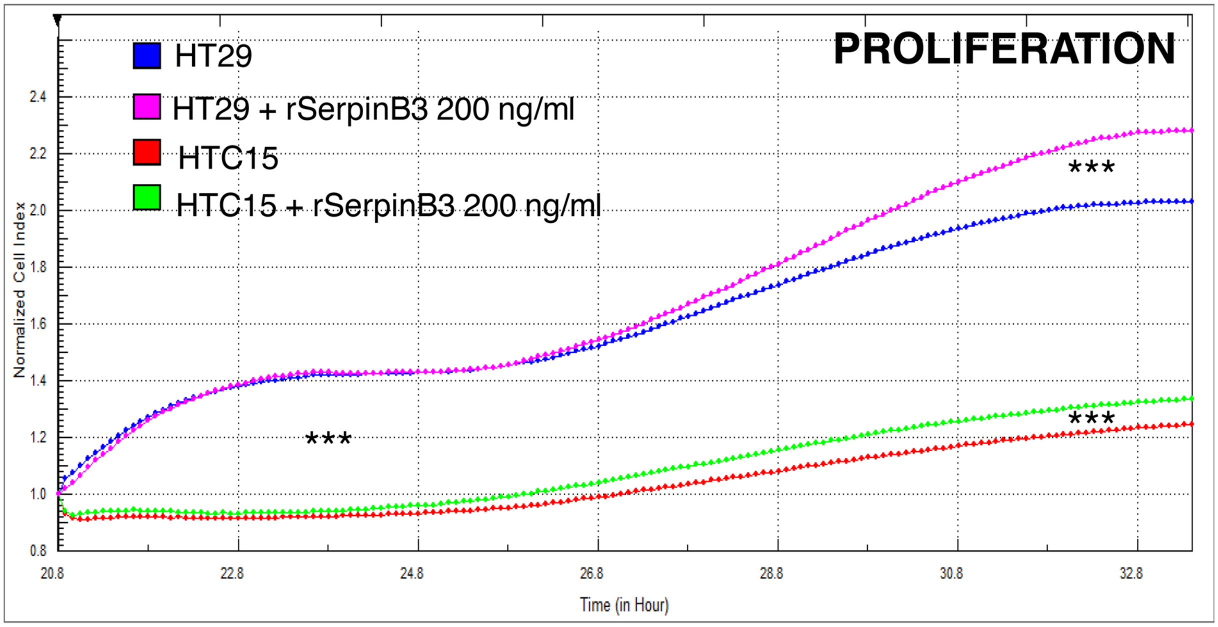

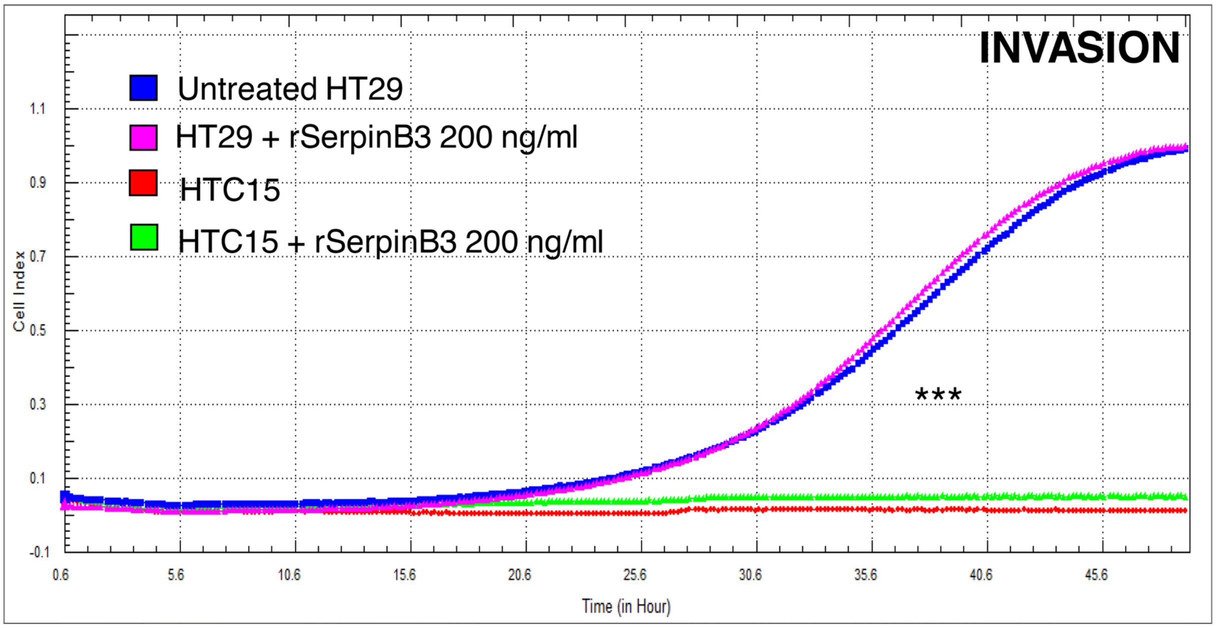

Supplementary Figure 5: Real time analysis of cellular proliferation and invasion in presence or in absence of recombinant SerpinB3 (rSerpinB3). In the upper panel HT29 and HT15 cellular proliferation, expressed as cell index, was measured as impedance-based signals that reflect cell adherence, using the xCELLigence RTCA (Real-time Cell Analyzer) DP (dual-plate) instrument. In the lower panel cellular invasion was assessed using the same system, where cells seeded in the upper chamber moved through the microporous membrane coated with Matrigel into the lower chamber containing fetal bovin serum as chemoattractant. rSerpinB3: recombinant SerpinB3. ***$p<0.0001$ (proliferation and invasion: HT29 vs HTC15; Proliferation: HT29 vs HT29 + rSerpinB3 and HTC15 vs HTC15 + rSerpinB3).

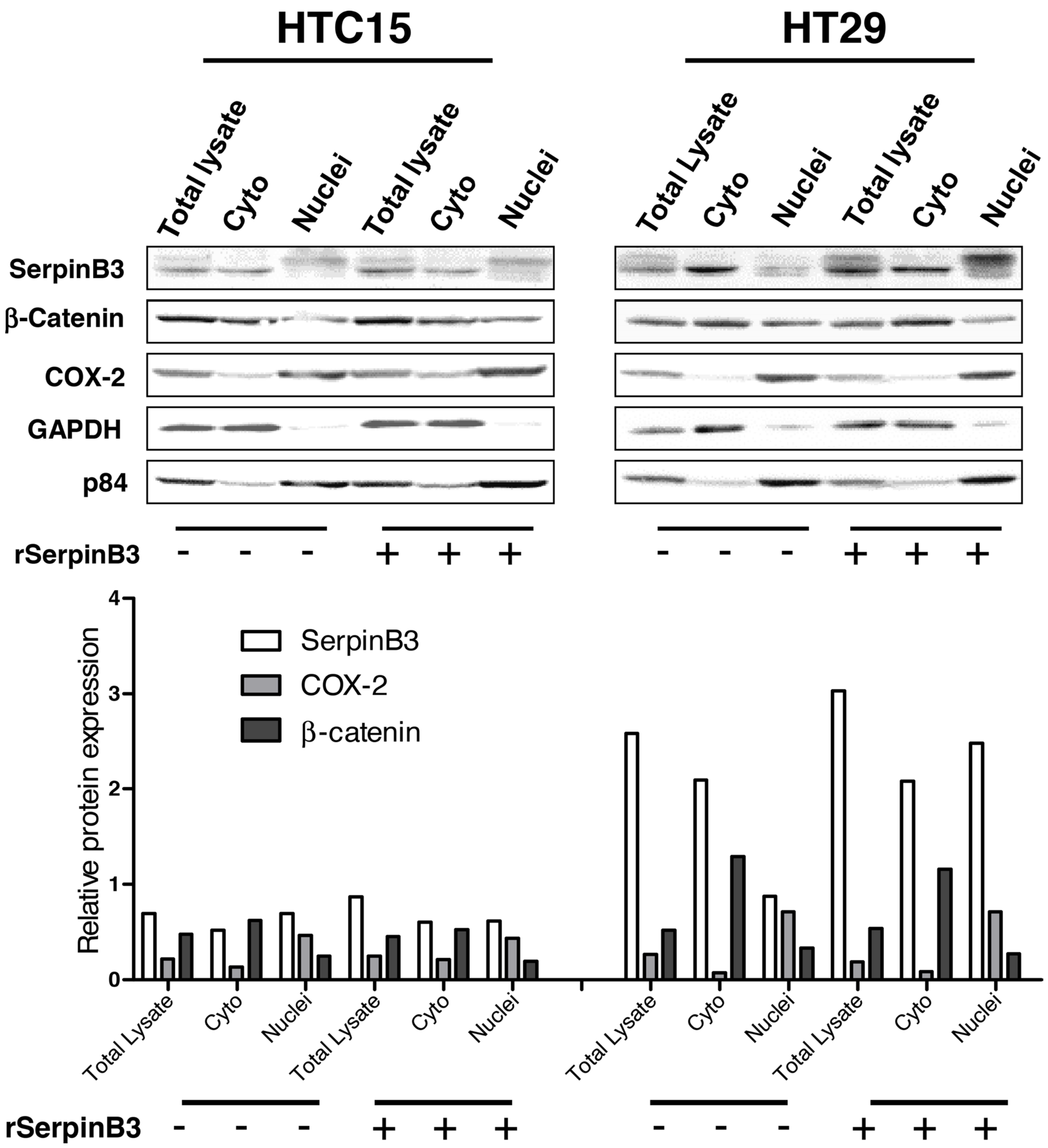

Supplementary Figure 6: Western blot analysis in cellular fractions. Western blot analysis of total lysates and of cellular fractions obtained from HTC15 and H29 cell lines after overnight incubation or not with recombinant SerpinB3 (rSerpinB3) at 200 ng/ml. In the lower panel the corresponding normalized densitometric analysis is reported.

Supplementary Table 1: Absolute quantification of SerpinB3, COX-2 and β-Catenin mRNA expression, normalized by the HPRT1 housekeeping gene, in relation to the degree of displasia in patients with adenoma

Patient code	Grade of displasia	SerpinB3	COX-2	β-catenin
SA74	Low grade	2.4	33.8	1.5
VA49	Low grade	2.0	5.2	18.8
CA70	Low and high grade	7.1	13.1	37.9
GG76	Low and high grade	17	29.7	1.1
TV76	High grade	23.5	26.8	291.1
